# New Hypothesis on Pathogenesis of Ovarian Cancer Lead to Future Tailored Approaches

**DOI:** 10.1155/2013/852839

**Published:** 2013-08-25

**Authors:** P. Rescigno, I. Cerillo, R. Ruocco, C. Condello, S. De Placido, M. Pensabene

**Affiliations:** Department of Clinical Medicine and Surgery, University Hospital and University Federico II, via Pansini 5, 80131 Naples, Italy

## Abstract

In the last decades, management of epithelial ovarian cancer (EOC) has been based on the staging system of the International Federation of Gynecology and Obstetrics (FIGO), and different classifications have been proposed for EOC that take account of grade of differentiation, histological subtype, and clinical features. However, despite taxonomic efforts, EOC appears to be not a unique disease; its subtypes differ for epidemiological and genetic risk factors, precursor lesions, patterns of spread, response to chemotherapy, and prognosis. Nevertheless, carboplatin plus paclitaxel combination represents the only standard treatment in adjuvant and advanced settings. This paper summarizes theories about the classification and origin of EOC and classical and new prognostic factors. It presents data about standard treatment and novel agents. We speculate about the possibility to create tailored therapy based on specific mutations in ovarian cancer and to personalize prevention.

## 1. Introduction 

Ovarian cancer is the seventh most common cancer in women worldwide (incidence) and the 2nd leading cause of death worldwide (mortality) among gynecological malignancies. However, malignant epithelial ovarian tumors (carcinomas) are the most lethal gynecological malignancies [1]. In the last decades, management of epithelial ovarian cancer (EOC) has been based on the staging system of the International Federation of Gynecology and Obstetrics (FIGO) and the evaluation of histological features of tumors [2]. Platinum-based schedules have represented the gold standard of cure, just recently improved with the introduction of bevacizumab in front line [3, 4]. Two studies showed better outcome particularly in progression-free survival (PFS) and a positive trend in overall survival (OS) with biological treatment [5, 6]. The most recent hypothesis on the pathogenesis of ovarian cancer introduces the concept of a different disease, opening new frontiers and scenarios for the future treatment [7, 8]. As for other cancers, in which the identification of specific biomarkers and biological features has led to target and tailored treatments, in future, the right treatment for the right patient could be chosen also for ovarian cancer.

Here, we reviewed the relevant clinical aspects of the theories on the pathogenesis of EOC and the potential implications by translating molecular research findings in preventive and treatment settings.

## 2. Ovarian Cancer: One Name for Different Diseases

The ovarian cancers may be distinguished, according to the grade of differentiation, from nuclear atypia and the presence or absence of stromal invasion into three groups: benign, borderline, and malignant tumors (carcinoma) [9]. The EOCs are a heterogeneous group of tumors that can be classified according to the histology in serous, mucinous, endometrioid, clear cell, and transitional and squamous tumors [9].

In order to explain the heterogeneity of EOC, Kurman and Shih proposed a new classification which divided ovarian carcinomas into two categories, type I and type II [7].

Type I includes low-grade serous, low-grade endometrioid, clear cell, and mucinous carcinomas. These cancers account for 25% of ovarian malignancies and cause 10% of deaths. These tumors frequently are diagnosed in early stage and have indolent behavior and good prognosis. These tumors are characterized by slow growth and appear as voluminous and unilateral masses. They are characterized by genetic stability, and frequent mutations have been described for KRAS, BRAF, PTEN, PIK3CA, and ERBB2. For type I ovarian carcinomas, morphological precursors have been identified as those leading to the development of invasive tumors by successive transformations.

Type II includes the high-grade serous, high-grade endometrioid, and undifferentiated carcinomas. They account for 75% of ovarian cancers and cause 90% of deaths. These tumors are diagnosed in advanced stage because of rapid growth [7]. They are characterized by genomic instability such as p53 mutation [10], inactivation of BRCA1/2 [11], and CCNE1 amplification.

According to Prat, the classification of ovarian cancer in just two types could be reductive from clinical point of view, so he divided EOC in five groups: high-grade serous (HGSC), endometrioid (ECs), clear cell (CCCs), mucinous (MCs), and low-grade serous (LGSC) [8]. Those variants represent distinct diseases, as supported by differences in epidemiology, genetic risk factors, molecular events, premalignant lesions, patterns of spread, response to chemotherapy, and prognosis [8].

HGSCs are the most common ovarian carcinomas (70%) and occur in the advanced stage and spread beyond the ovary at diagnosis. They exhibit p53, BRCA,WT1, and p16 mutations and high ki67 levels and frequently express estrogen receptors (ERs). ERs are also expressed in LGSCs and ECs, but they are negative in almost all CCCs and MCs. 

LGSCs account for <5% of all cases of EOC, are frequently associated with a serous borderline tumor and follow a relatively indolent course. They show KRAS and BRAF mutations but not BRCA and p53 alterations.

MCs are 3-4% of ovarian tumors, show gastrointestinal differentiation, large size, and unilaterality, and are usually confined to the ovary. KRAS mutations are an early event in mucinous tumorigenesis. These tumors are also frequently immunoreactive for cytokeratin 7 and 20.

ECs represent 10% of all ovarian carcinomas. They occur more frequently in perimenopausal age and at an early stage. These tumors are bilateral in 28% of cases, are associated with 15–20% of cases with endometrium carcinomas, and seem to arise from endometriotic cysts. High-grade ECs are morphologically indistinguishable from HGSCs, and they often express WT1. ECs are characterized by ARID1A mutations and CTNNB abnormalities and are associated with favorable outcome and PTEN inactivation or PIK3CA mutations; ECs are also immunoreactive for vimentin, cytokeratins 7 and 20, ERs, and progesterone receptors.

CCCs account for 10% of ovarian carcinomas, and patients typically present early stage disease. They are associated with endometriosis and show unfavorable prognosis when they are present at advanced stage. These tumors carry ARID1A mutations and are usually positive for HNF1-*β* [8].

## 3. Ovarian Cancer: What Origin?

**Figure 1:**
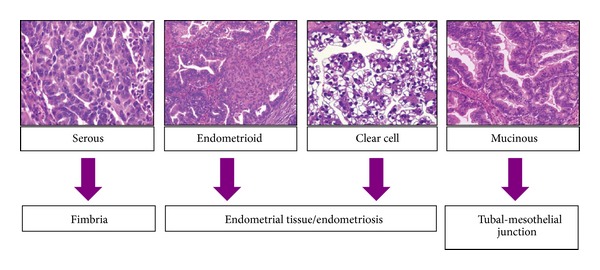
Pathogenesis of different EOCs according to Prat.

Traditional theory led back the origin of EOC to ovarian surface epithelium (mesothelium), and subsequent metaplastic changes lead to the development of the different cell types (serous, endometrioid, clear cell, mucinous, and transitional cell) [12]. Recent theory introduced the concept that the different histotypes of EOC originated from three different sites. In Figure 1, the origin of EOC from fimbria, endometrial tissue, and tubal-mesothelium junction is depicted. According to this theory, serous ovarian cancer originates from the fallopian tubes [13].

In carriers of BRCA mutation, a lesion similar to a serous tubal intraepithelial carcinoma (STIC) [14] has been described. Therefore, adnexal mass could originate from implantation of tubal tumor cells to the ovary [15]. Many studies have shown that the STIC and small invasive cancers can be recognized not only in women with a genetic predisposition but also in 50–60% of sporadic ovarian cancers [16]. The first transformation process seems to take place in the secretory cells of tube [17]. In addition, p53 mutations have been identified in the STIC synchronous with high-grade serous carcinomas. A recent study on the genetic expression of high-grade serous carcinomas also showed a higher correlation with the tubal epithelium than with the ovarian surface epithelium [7]. 

Dysplastic and hyperplastic lesions were frequently found in fallopian tubes in BRCA1/2 mutation carriers that underwent prophylactic surgery. Such lesions showed changes in cell cycle and apoptosis related proteins suggesting a premalignant phenotype. Moreover, in this setting, occult carcinomas have been reported in 2–11% of the ovaries removed. The majority of these occult carcinomas are seen within the fallopian tube, especially in the fimbriae, which has led to the hypothesis that many BRCA-associated ovarian cancers may have originated in the fallopian tube [19, 20].

A condition was then described in which the tubal cells are apparently normal with an overexpression of p53, defined “*p53 signature*” [21]. This is comprised exclusively of secretory cells, and the majority exhibit evidence of DNA damage. About 57% contain p53 mutations with a low Ki67 proliferation index. It is unclear if the p53 signature represents a premalignant lesion or a benign overexpression of p53 to DNA damages without biological relevance [7]. p53 mutation is an early event in the genesis of HGSC, occurring in p53 signature foci and leading to STIC in the distal fallopian tube. BRCA1 mutations also occur early during the development of STIC but after p53 mutation [22].

LGSCs originate from a noninvasive serous borderline tumor. They represent the progression of this precursor beyond microinvasion. The presence of small foci of LGSC and ovarian borderline tumor is associated with an excellent prognosis. Advance stage LGSCs are less favorable; nevertheless, the disease usually follows a relatively indolent course [7]. 

ECs and low-grade CCCs originate from endometriotic cysts and are frequently associated with implantation of endometriosis in the pelvis [22]. The origin of endometriosis has not yet been clarified. It is unclear whether it is related to a process of metaplasia or flow retrograde menstrual. The latter hypothesis is the most reasonable suggesting that ECs and CCCs develop from the endometrial tissue implanted on the ovary. Endometriosis shows molecular abnormalities, including the activation of the oncogenetic pathway [23] that allows the endometrial tissue to implant, survive, and invade the ovarian and peritoneal tissue. The tubal ligation seems to have a protective effect on the development of endometrioid and clear cell carcinoma because this procedure prevents the retrograde menstrual flow, while it does not prevent high-grade serous carcinomas because it does not avoid the possible exfoliation of abnormal tubal cells [7].

The primary MCs of the ovary are rare, accounting for 3% of EOC. The origin of these tumors is not clear because it is not possible to recognize a Müllerian phenotype. Although it has been suggested that they may have a similar origin to endocervical tumors, the epithelium that characterizes the MCs is more similar to the gastrointestinal mucosa. An association between MCs and tumors of Brenner (transitional cell) has been described. They show the “Walthard cell nest's” elements consisting of a benign transitional epithelium, which have been found in the paraovarian and paratubal cysts. Therefore, the MCs may originate from transitional cell at the tube-peritoneal junction [7].

## 4. Prognostic and/or Predictive Factors Are Not a Shared Consensus

Histologic subtype, tumor grade, and disease stage are currently used to stratify patients into high versus low-risk disease and predict response to therapy. These features allow the clinicians to divide early EOCs in two risk classes: low risk (stage IA-B, G1-2) and high risk (stages IA-B, G3, and IC-II, clear cell or undifferentiated histology) [24]. However, EOC is diagnosed at an early stage (I and II FIGO stages) only in about 25% of the cases; more frequently (70% of cases), the diagnosis is posed at late stages. The five-year survival of these patients is 70–90% in stage I, 50–60% in stage II, 20–40% in stage III, and 10% in stage IV [25].

A large series study, conducted on 575 women affected by EOC, optimally surgically debulked without macroscopic residual disease, showed that tumor cell type was both more reproducible and provided superior prognostic information compared with assignment of tumor grade [26, 27]. Tumor-cell type was confirmed to be the most relevant histopathological prognostic also in patients treated with surgery and chemotherapy [28]. MCs and CCCs have been associated with inferior outcome [29–31].

Significance of tumor grade remained only for low- versus high-grade serous carcinomas, while grade 2 and 3 tumors had a similar prognosis. Tumor grade prognostic significance is irrelevant in MCs, ECs, and CCCs subtypes [32]. 

EOCs appear as a heterogeneous disease. DNA or cellular alterations that lead tumor behaviour, not routinely detected, should represent new prognostic or predictive factors.

The cell cycle is regulated by two major families of cyclin kinase inhibitors (CKIs). p21 and p27 are members of Cip/kip family and inhibit cyclin E/CDK [33] arresting cells in G1 phase.

Wild type p53 is a negative regulator of cell cycle progression and works on activating p21 expression. It also induces cells to apoptosis when DNA damages are revealed [34]. Tumor protein 53 (p53) alterations are present in 96% of HGSCs but are rare in LGSCs [35].

Mutations of the p21 and p27 genes occur less frequently in human cancer than p53 alterations [36]. Immunostaining (IHC) of p21 alone [37], or concomitant loss of p21 (negativity), overexpression of p53 (positivity) [38], and the combination of low p21 and p27 expression were associated with worse overall survival [39] for EOC in several studies. In a recent study, p53 and p21 status in 129 ovarian cancers was not related to serous/nonserous tumors, tumor grade, or FIGO stage. However, the p53 status was significantly associated with disease-free survival (DFS) in a univariate analysis. Patients with p53 negative tumors had a 5-year DFS of 82% compared with 62% in p53 positive tumors [40].

The role of HER2 in EOC is still unclear. HER2 overexpression is associated with poor prognosis, and ovarian cancers are estimated to be HER2 positive in 10% of cases [41]. Up-to-date there is no consensus about the evaluation protocol applied for HER2 overexpression in EOC. New tools for the detection of HER2 in ovarian cancer have been evaluated such as real-time polymerase chain reaction (PCR) analysis on cancer cells captured by laser microdissection in order to optimize the identification of patients overexpressing HER2 that could benefit from a target therapy with trastuzumab or pertuzumab [42].

Amplicon-dependent expression of the cell cycle protein cyclin E (CCNE1) has been recently detected as predictor of survival in advanced EOC [43]. CCNE1 amplification represents an oncogenic stimulus through the activation of the cell cycle and contributes to primary resistance to cisplatin. Moreover, CCNE1 gene amplification is also a predictive biomarker of therapeutic response for several new targeted therapeutic options, as BMS-387032, P1446A-05, flavopiridol, and seliciclib [36].

BRCA1 and BRCA2 mutations, caused by germline or somatic alterations, play a key role in ovarian carcinogenesis. The BRCA genes encode proteins that enter in mismatch repair systems, particularly homologous recombination, repairing DNA double helix damage [44]. BRCA1 or BRCA2 mutations are a high-risk factor for HGSC, and women harboring such mutations have a 30% to 70% probability of developing ovarian cancer by the age of 70 [45, 46].

Incidence of germline or somatic mutations in BRCA1/2 genes is up to 22% in HGSC. BRCA1/2 function can be lost also through DNA hypermethylation or epigenetic silencing (*BRCAness phenotype*), but these mechanisms are mutually exclusive of either BRCA1 or BRCA2 mutations [36]. Defects in homologous-recombination repair can also be caused by loss of function of proteins other than BRCA1 and BRCA2, including the RAD51, ataxia telangiectasia mutated (ATM), ataxia telangiectasia and Rad3 related (ATR), and checkpoint kinase 1 and 2 homologue (CHK1 and CHK2) proteins, as well as components of the Fanconi's anemia repair pathway [44]. Loss of function of these proteins also sensitizes cells to PARP inhibition. Such defects in homologous-recombination repair may be relatively common in some sporadic ovarian cancers [47], potentially making this therapeutic strategy more widely useful as an anticancer treatment. Only one wild allele of BRCA1 or BRCA2 is sufficient for DNA repair mechanism, so as in Knudson's model, and additional somatic loss of the wild-type allele is necessary to develop ovarian carcinoma in women with a germline BRCA mutation [48]. Furthermore, when BRCA is mutated, homologous recombination does not work and DNA damage cannot be repaired. Carboplatin acts just for creating DNA double helix damage, which justifies BRCA mutant patients better prognosis compared to sporadic forms [49].

TP53 mutations and ovarian cancers with loss of BRCA1 and/or BRCA2 function seem to be target for new therapies as inhibitors of poly(ADP-ribose) polymerase (PARP). So that detecting BRCA mutations should be predictor of therapy response [50].

K-RAS is a member of family able to regulate cell growth, survival, and differentiation activating downstream effectors [51]. Mutations in KRAS gene are typical of low-grade ovarian tumors while are rare in III and IV stages [52]. Other mechanisms able to deregulate KRAS gene in ovarian cancer are gene amplifications that account in about 11% of ovarian tumors [53] and short RNA molecule (miRNA) let-7 [54]. Let-7 binds to its specific site in the 3′-UTR of KRAS mRNA and induces KRAS downregulation [54]. Recently, KRAS-LCS6 polymorphism has shown to be not relevant in ovarian cancer and not associated to any outcome or physiopathological characteristic [55].

Mutations in the p110 subunit of PI3K, called PIK3CA, are often responsible for activation of the phosphatidylinositol 3-kinase (PI3K)/AKT/mammalian target of rapamycin (mTOR) pathway and have been detected in 12% of ovarian cancers [56]. Several preclinical studies suggested that PIK3CA mutations could predict response to PI3K and mTOR inhibitors, although mutations in the mitogen-activated protein kinase (MAPK) pathway (KRAS, NRAS, BRAF) might mediate resistance [57, 58].

In a recent study, twenty-three PIK3CA-mutant women, after a first-line standard therapy, were treated with an inhibitor of this pathway showing a higher response rate (30%) compared to patients under standard treatment (10%) independently from their primary malignancies, that is, breast or gynaecological cancers [59].

Mutations in BRAF and KRAS, components of the mitogen-activated protein kinase (MAPK) cascade, are common in LGSCs and serous borderline ovarian tumors, but they are present in <1% of HGSCs. So that patients with BRAF mutations may have an improved clinical outcome [60].

However, serous borderline and LGSCs commonly are chemotherapy-resistant diseases, with response rates to chemotherapy of 4% in the neoadjuvant setting and 2.1%–4.9% in the recurrent setting [61]. 

Given the high prevalence of BRAF and KRAS mutations in these tumors, there has been recent interest in testing inhibitors targeting the MAPK pathway in patients with advanced disease. Nevertheless, patients with aggressive LGSCs do not harbour BRAF mutation typically. Moreover, vemurafenib, a selective RAF inhibitor, lacks his activity in KRAS mutant and BRAF/RASWT tumors, so it could have a limited utility in this setting of patients [62].

On the contrary, a recent phase 2 trial of the MEK (MAP kinase kinase) inhibitor AZD6244 in women with recurrent LGSCs of the ovary or peritoneum reported a radiographic response rate of 15.4% [63].

A recent trial has shed new light on this regard. It demonstrated that BRAF mutations are common in surgically treated patients who did not recur but are rarely present in patients who required systemic therapy. It suggested that highly selective RAF inhibitors may have limited utility in this disease; nevertheless, a subset of LGSCs depends on MEK activity [64].

ARID1A is a mutated gene in a significant portion of CCCs and ECs, but it is not detected in serous ovarian tumors [18]. The ARID1A gene encodes a member of the SWI/SNF family, BAF250a, that is able to regulate gene transcription altering chromatin structure [18]. It is detectable in atypical endometriosis, suggesting that this is an early event in a multistep carcinogenesis of ECs. Recently, loss of BAF250a was reported in almost half of ECS and CCCs, but no association was found between BAF250a expression and any clinical and pathological risk factors or survival.

EOCs are associated with high levels of vascular endothelial growth factor (VEGF), a protein related to tumor growth and metastatic process. VEGF is correlated with worse outcome of EOCs patients [65].

## 5. Conventional Treatments of EOC 

The therapy for stage I is undoubtedly total abdominal hysterectomy and bilateral salpingo-oophorectomy with careful surgical staging. Pelvic and periaortic nodes may be involved in 10–20% of the time in apparent stage I disease, and lymphadenectomy is considered an important diagnostic and therapeutic procedure [3].

Adjuvant chemotherapy is indicated in early stages except for low risk (stage IA-B, G1-2) EOCs. ICON 1 trial showed an increase in PFS and OS in patients who received platinum-based adjuvant treatment, especially for the high-risk group (G3 IA, IB, IC G2-G3, clear cell) [66].

The combination of carboplatin and paclitaxel has represented the first-line standard treatment more for early than for advanced EOC, as demonstrated by GOG 111 [67] and OV 10 trials [68]. Just recently, the association of bevacizumab has gained better PFS. Among the most promising targets identified in ovarian cancer, a leading role belongs to angiogenesis. In recent years, several phase II studies have demonstrated the toxicity profile and activity of bevacizumab, a monoclonal recombinant antibody that binds VEGF-A, in the treatment of relapsed ovarian cancer [69, 70]. Two recent phase III trials, that is, the GOG218 [5, 6] and ICON7, have shown the efficacy of bevacizumab associated to standard treatment, in prolonging PFS of about four months compared to chemotherapy alone (10.7 versus 14.1 months). A preliminary analysis has indicated a trend in improving OS. These findings led the European Medicines Agency (EMA) to the approval of bevacizumab in combination with carboplatin and paclitaxel as upfront treatment in the in advanced EOCs, peritoneal cancers, and tubal cancers. 

Despite the availability of a fist-line single treatment, the different subtypes of EOCs showed dissimilar outcome to chemotherapy. CCCs and MCs are less likely to respond to chemotherapy, but particularly CCCs show a lower 5-year survival in late stage than HGSCs (20% versus 30%) [71, 72]. Indeed, whereas highly proliferative cells of HGSC show sensitivity to platinum due to loss of the ability to repair double-stranded DNA, the less proliferative CCCs are less sensitive to platinum compounds because of their more genetically stable cells [73].

Recent studies demonstrated greater sensitivity to platinum based chemotherapy for patients with p53 mutation, in contrast to previously believed [74].

As well as p53 mutations, BRCA deficient cells show a higher sensitivity to platinum compound in preclinical setting [75]. In clinical setting, Bolton et al. have shown an improved 5-year OS in BRCA1 and BRCA2-related EOCs at a standard treatment, despite a later stage and higher grade at diagnosis. The 5-year OS was 36% for noncarriers, 44% for BRCA1 carriers, and 52% for BRCA2 carriers. After adjusting for study and year of diagnosis, BRCA1 and BRCA2 mutation carriers showed a more favourable survival than noncarriers. These survival differences remained after additional adjustment for stage, grade, histology, and age at diagnosis. BRCA2 carriers had the best prognosis [76].

## 6. Novel Agents 

Novel agents are under investigation in EOCs. In particular, genetic mutations can be used as molecular targets for new selective pharmacological agents.

Different chemotherapeutic agents, such as liposomal doxorubicin and trabectedin, are observed to have higher response rates in patients with BRCA mutation or with *BRCAness phenotype* [77].

In this setting, also PARP inhibitors have been studied. PARPs are multifunctional enzymes that play an important role in the repair of DNA single-strand breaks. The inhibition of PARPs causes the accumulation of DNA single-strand breaks leading to DNA double-strand breaks. Normal cells are able to repair this damage by homologous recombination, but in BRCA1/2 mutation carriers, these lesions are not repaired resulting in cell cycle arrest and cell death. Farmer et al. demonstrated that the decreasing PARP1 expression with RNA expression interference resulted in the reduction of the survival of BRCA1/2 embryonic stem cells compared with wild-type cells [78]. The first-in-human clinical trial of the PARP inhibitor, olaparib, has been conducted in patients with BRCA1/2-mutated advanced cancers including ovarian, breast, and prostate cancers [79]. Olaparib demonstrated a very acceptable side effect profile when compared with conventional chemotherapies. A durable antitumor activity was found in cancer associated with the *BRCA1 *or *BRCA2 *mutation. These data indicate that using PARP inhibition to target a specific DNA-repair pathway has the necessary selectivity profile and a wide therapeutic window for *BRCA*-deficient cells, supporting the clinical relevance of the hypothesis that *BRCA *mutation-associated cancers are susceptible to a synthetic lethal therapeutic approach [80, 81]. 

In the ICEBERG2 study, Audeh et al. provided positive proof of the concept of the efficacy and tolerability of genetically targeted treatment with olaparib in *BRCA*-mutated advanced ovarian cancer [82]. Activity of olaparib was observed both in platinum-sensitive and platinum-resistant patients, suggesting that resistance mechanisms to olaparib might only partly overlap with those for platinum chemotherapies. Although phase III trials in EOC are not available, in triple negative breast cancer, a negative phase III trial opened the concept of a better understanding of mechanisms supporting PARP resistance and a better definition of clinical setting for PARP inhibitors development [83].

A recent analysis in patients with BRCA1/2-mutated ovarian cancer has suggested that chemosensibility, particularly with carboplatin and taxanes, is maintained in such patients after disease progression on olaparib [84].

Kristjansdottir and Dizon have recently reviewed the role of HER-dimerization inhibitors, in particular evaluating the scientific rationale of pertuzumab in ovarian cancers. To date, pertuzumab is the most extensively studied HER2 inhibitor in ovarian cancer, with almost 400 patients having been evaluated in phase II studies. Pertuzumab showed limited activity in platinum-sensitive and platinum-resistant disease in recurrent ovarian cancer when combined with chemotherapy. However, a subset analysis suggests that pertuzumab has a high response in the subgroup of patients with activated HER2 or low HER3 mRNA expression. Therefore, HER2 activation and HER3 mRNA levels may predict response to pertuzumab in ovarian cancer [85]. Further prospective biomarker-led trials are warranted.

A large phase II study concluded that the clinical value of single-agent trastuzumab in recurrent ovarian cancer is limited by the low frequency of HER2 overexpression and low rate of objective response among patients with HER2 overexpression [86]. 

The negative findings of the previous studies can be explained by the hypothesis that other molecular pathways interfere with the EGFR/HER2 signalling, such as follicle stimulating hormone receptor (FSHR), potentially minimizing HER2 impact on ovarian cancer cell proliferation and ultimately to the HER2 effect on disease progression and prognosis. HER2 can be a negative prognosticator only in FSHR negative EOC cases [87].

## 7. Implications for Screening, Prevention, and Treatment

Early detection of EOC has represented a challenge in the last decades. Pelvic examination, transvaginal ultrasound, and detection of serum CA125 are current measures in cancer screening, without reaching evidence of good outcome [24]. The dualistic model of the pathogenesis of EOC suggests the need of different approaches for prevention of different types of EOCs. Type I tumors (low-grade serous, low-grade endometrioid, clear cell, and mucinous) are slow growing and reach a large size while still confined to the ovary [7]. They could be detected by pelvic examination and/or transvaginal ultrasound. The early diagnosis of type II EOC is a more complex challenge. Type II tumors (high-grade serous, undifferentiated carcinomas, and malignant mixed mesodermal tumors) originate outside the ovary [7] and suggest new screening approaches, by developing a panel of sensitive and specific biomarkers that are expressed early in ovarian carcinogenesis.

Prophylactic salpingo-oophorectomy is currently recommended as strategy to reduce ovarian cancer risk [88] in BRCA1 and BRCA2 mutation carriers. As in the PROSE consortium study [89], prophylactic surgery significantly reduces ovarian cancer (HR = 0.31; 95% CI = 0.12–0.82) and breast cancer risk when performed in women under 50 years, especially in BRCA2 mutation carriers (HR = 0.36; 95% CI = 0.16–0.82) than in BRCA1 (HR = 0.63; 95% CI = 0.41–0.96). At a median followup of about 5 years, breast (HR = 0.44; 95% CI = 0.26–0.76) and ovarian cancer specific (HR = 0.25; 95% CI = 0.08–0.75) mortality rates were reduced. Premature or precocious menopause has been associated with long-term effects [90], that is, increased cardiovascular disease, cognitive impairment, psychiatric symptoms, impaired sexual function, osteoporosis, and bone fractures. The recent hypothesis on the pathogenesis of ovarian cancer BRCA-correlated from fimbria and the subsequent implants on the surface of the ovaries could modify prophylactic strategies limiting the surgery to tubes, sparing the endocrine function of the ovaries, and avoiding the short- and long-term effects on women's health of such risk management option.

As with early detection, the treatment of type I and type II tumors must be individualized. Type I tumors are generally low grade, slow growing, and localized to the ovary at diagnosis, spreading late in their evolution. Accordingly, when confined to the ovary, conservative surgery, that is, salpingo-oophorectomy, could be a sufficient approach. On the other hand, when an adjuvant treatment is required according to stage, different schedules could be improved for type I and type II tumors, in agreement of the peculiar biomolecular profile. In type I EOC, it is reasonable to develop in clinical setting BRAF inhibitors and other MAPK kinase inhibitors in order to improve progression-free survival and overall survival in patients with advanced EOC [62], actually treated with conventional chemotherapeutic schedules. 

In Table 1, Prat theory has been summarized with a focus on the clinical implications about the potential drugs to be used for the five different subtypes of EOC.

On the other hand, type II EOC, currently identified as EOC with a germline or acquired mutations in BRCA genes, as well as sporadic ovarian cancer with a *BRCAness phenotype* [91], could benefit the recognition of molecular mechanisms involved in PARP pathway. The potential benefit of PARP inhibitors might be maximized in type II ovarian cancer. It will be crucial to explore novel therapeutic trial strategies and drug combinations, including PARP inhibitors, and incorporate robust biomarkers predictive of response if these drugs are to reach their full potential. 

In Table 2, ongoing clinical trials on target therapies in EOC are summarized. Trials have been selected among studies registered at the canver.gov website [92].

## 8. Conclusion

Regardless of dualistic or five subtypes hypothesis, the molecular characterization of ovarian cancer in different subtypes is the cornerstone to build a personalized approach in the era of personalized medicine.

## Figures and Tables

**Table 1:** The correlation of the five types of Prat theory and clinical implications.

	HGSH	LGSH	MC	EC	CCC
Prat theory					
Precursor lesions	STIC	Serous borderline tumor	Cystoadenoma/borderline tumor	Atypical endometriosis	Atypical endometriosis
Pattern of spread	Very early transcoelomic spread	Transcoelomic spread	Usually limited to ovary	Usually limited to pelvis	Usually limited to pelvis
Molecular abnormalities	BRCA P53	BRAF KRAS	KRAS HER2	PTEN ARID1A	HNF1 ARID1A
Chemosensitivity	High	Intermediate	Low	High	Low
Prognosis	Poor	Intermediate	Favorable	Favorable	Intermediate

Clinical implications					
Potential target therapies	PARP inhibitors	BRAF KRAS inhibitors	Monoclonal antibodies Anti-HER2 KRAS inhibitors	Not Available	Not available
Potential role of screening	Research of novel early biomarkers	Research of novel early biomarkers	Transvaginal ultrasound + CA125	Transvaginal ultrasound + CA125	Transvaginal ultrasound + CA125

HGSC: high-grade serous carcinoma; LGSC: low-grade serous carcinoma; EC: endometrioid carcinoma; CCC: clear cell carcinoma; MC: mucinous carcinoma; STIC: serous tubal intraepithelial carcinoma.

**Table 2:** Ongoing clinical trials on target therapies in epithelial ovarian cancer.

Agent	Study	Phase	Protocol IDS
PARP inhibitors			
Olaparib	Cediranib and Olaparib in Combination for Recurrent Ovarian or Triple-Negative Breast Cancer	I/II	DFCI 09-293
EE7449	An open-label, multicenter, phase 1/2 study of poly(ADP-Ribose) polymerase (PARP) inhibitor E7449 as single agent in subjects with advanced solid tumors or With B-cell malignancies and in combination with temozolomide (TMZ) or with carboplatin and paclitaxel in subjects With advanced solid tumors	I/II	E7449-E044-101
Olaparib	Phase Ib study of olaparib plus weekly carboplatin and paclitaxel in relapsed ovarian cancer	I/II	ISS22810034
Veliparib	Veliparib and topotecan for relapsed ovarian cancer with negative or unknown BRCA status	I/II	VeTo
Rucaparib	Rucaparib(CO-338; formally called AG-014699 or PF-0136738) in treating patients with locally advanced or metastatic breast cancer or advanced ovarian cancer	II	CDR0000593558
Veliparib	Veliparib and topotecan hydrochloride in treating patients with solid tumors, relapsed or refractory ovarian cancer, or primary peritoneal cancer	I/II	NCI-2011-00312
Veliparib	Veliparib monotherapy for relapsed ovarian cancer with BRCA mutation	I/II	Veli-BRCA
Veliparib	Veliparib and floxuridine in treating patients with metastatic epithelial ovarian, primary peritoneal cavity, or fallopian tube cancer	I	NCI-2012-02767
BKM120/olaparib	Phase I of BKM120/olaparib for triple negative breast cancer or high-grade serous ovarian cancer	I	12-159
ABT-888	ABT-888 in treating patients with malignant solid tumors that did not respond to previous therapy	I	NCI-2011-01472
ABT-888	Carboplatin, paclitaxel, bevacizumab, and ABT-888 in treating patients with newly diagnosed stage II, stage III, or stage IV ovarian epithelial cancer, fallopian tube cancer, or primary peritoneal cancer	I	NCI-2011-03730
ABT-767	Study of ABT-767 in subjects with breast cancer 1 and breast cancer 2 (BRCA 1 and BRCA 2) mutations and solid tumors or high-grade serous ovarian, fallopian tube, or primary peritoneal cancer	I	M10-976
Veliparib	Veliparib, paclitaxel, and carboplatin in treating patients with solid tumors that are metastatic or cannot be removed by surgery and liver or kidney dysfunction	I	NCI-2011-02500
Veliparib	Veliparib and liposomal doxorubicin hydrochloride in treating patients with recurrent ovarian cancer, fallopian tube cancer, primary peritoneal cancer, or metastatic breast cancer	I	CDR0000674917
Veliparib	Phase II study of veliparib in patients with persistent or recurrent epithelial ovarian, fallopian tube, or primary peritoneal cancer who carry a germline BRCA 1 or BRCA 2 mutation	II	GOG-0280
Veliparib	Veliparib, oxaliplatin, and capecitabine in treating patients with advanced solid tumors	I	NCI-2011-02543
Olaparib	Olaparib in combination with carboplatin for refractory or recurrent women's cancers	I	110022
Veliparib	Veliparib, doxorubicin hydrochloride, and carboplatin in treating patients with recurrent ovarian cancer, primary peritoneal cancer, or fallopian tube cancer	I	NCI-2011-01977
AZD2281	AZD2281 plus carboplatin to treat breast and ovarian cancers	I	080092

VEGF inhibitors			
RAD001 + Bevacizumab	Study of RAD001 and bevacizumab in recurrent ovarian, peritoneal, and fallopian tube cancers	II	09-01-RAD001BEV
VB-111 (ANTI VEGF)	A phase I/II trial of VB-111 and paclitaxel for recurrent platinum-resistant müllerian cancer	I/II	VB-111-157
Dalantercept	Phase II trial of dalantercept to treat ovarian, fallopian tube, or primary peritoneal cancer	II	GOG-0170R
Endostar	Endostar and/or cisplatin for treatment of malignant pleural effusion or ascites	II	SIM-90
Pazopanib	A randomized study of safety and efficacy of pazopanib and gemcitabine in persistent or relapsed ovarian cancer	II	16153
Pazopanib	Phase I clinical and pharmacokinetic study of pazopanib in a population of frail elderly patients according SIOG criteria	I	11GENE06
Pazopanib	Weekly paclitaxel with or without pazopanib in platinum-resistant or refractory ovarian cancer	II	MITO-11
Pazopanib	A phase I/II study of pazopanib (GW786034) and cyclophosphamide in patients with platinum-resistant recurrent, pretreated ovarian cancer	I/II	3107000
Pazopanib	Pazopanib hydrochloride, paclitaxel, and carboplatin in treating patients with refractory or resistant ovarian epithelial cancer, fallopian tube cancer, or peritoneal cancer	I/II	CDR0000703686
Pazopanib	Pazopanib and weekly topotecan in patients with recurrent ovarian cancer (TOPAZ)	I/II	PazTo_2010
Pazopanib	Paclitaxel with or without pazopanib hydrochloride in treating patients with persistent or recurrent ovarian epithelial, fallopian tube, or peritoneal cavity cancer	II	NCI-2011-03635
BIBF1120	Doxorubicin + BIBF 1120 in patients with ovarian cancer	I/II	GYN10-149
BIBF1120	Daily low dose chemotherapy and BIBF 1120 in advanced ovarian cancer	II	UCL/10/0470
BIBF 1120	BIBF 1120 in bevacizumab-resistant, persistent, or recurrent epithelial ovarian cancer	II	Pro00033060
BIBF1120	BIBF 1120 + carboplatin/pegylated liposomal doxorubicin (PLD) in patients with ovarian cancer (OC)	I	1199.119

mTOR inhibitors			
Temsirolimus	A study of combination of temsirolimus (Torisel) and pegylated liposomal doxorubicin (PLD, Doxil/Caelyx) in advanced or recurrent breast, endometrial, and ovarian cancer	I	UMCNONCO200902
Sirolimus	Sirolimus and vaccine therapy in treating patients with stage II–IV ovarian epithelial, fallopian tube, or primary peritoneal cavity cancer	I	I 199911
Everolimus	Carboplatin, Pegylated Liposomal Doxorubicin Hydrochloride, and Everolimus in Treating Patients With Relapsed Ovarian Epithelial, Fallopian Tube, or Peritoneal Cavity Cancer	I	IRB 10-019
Temsirolimus	Temsirolimus, carboplatin, and paclitaxel as first-line therapy in treating patients with newly diagnosed stage III or stage iv clear cell ovarian cancer	II	NCI-2011-02653
Everolimus + bevacizumab	Bevacizumab with or without everolimus in treating patients with recurrent or persistent ovarian epithelial cancer, fallopian tube cancer, or primary peritoneal cancer	II	CDR0000640439
Temsirolimus + bevacizumab	Temsirolimus and bevacizumab in treating patients with advanced endometrial, ovarian, liver, carcinoid, or islet cell cancer	II	NCI-2012-02086
Temsirolimus	Activity, tolerability, and safety of temsirolimus in women with ovarian cancer who progressed during previous platinum chemotherapy or within 6 months after therapy or advanced endometrial carcinoma	II	AGO-GYN 8
Temsirolimus	A Phase II study of sunitinib or temsirolimus in patients with advanced rare tumors	II	I206

Anti-HER2/Anti-EGFR			
Lapatinib	Lapatinib and paclitaxel in treating patients with advanced solid tumors	I	UCSF CC#05591
TCMC-Trastuzumab	Safety study of ^212^Pb-TCMC-trastuzumab radio immunotherapy	I	AREVAMED01
Pertuzumab	A study of pertuzumab in combination with standard chemotherapy in women with recurrent platinum-resistant epithelial ovarian cancer and low HER3 mRNA expression	II	MO28113
MGAH22 Anti HER2	Safety study of MGAH22 in HER2-positive carcinoma	I	CP-MGAH22-01
Panitumumab	Carboplatin-based chemotherapy with or without panitumumab in platinum-sensitive recurrent ovarian cancer	II	GMIHO-008/2009_AG56
Panitumumab	Panitumumab and gemcitabine in relapsed ovarian cancer	II	WIH 20050782
OSI906 IGFR inhibitor	A study evaluating intermittent and continuous OSI-906 and weekly paclitaxel in patients with recurrent epithelial ovarian cancer (and other solid tumors)	I/II	OSI-906-202

Kinase inhibitors			
Sorafenib	Efficacy and safety study of sorafenib with topotecan in patients with platinum-resistant recurrent ovarian cancer	II	TRIAS 2009
Sorafenib	Sorafenib and bevacizumab to treat ovarian, fallopian, and peritoneal cancers	II	070058
Sorafenib	Oral Hydroxychloroquine Plus Oral Sorafenib to Treat Patients With Refractory or Relapsed Solid Tumors	I	CTRC 11-71
Sunitinib	Sunitinib and hydroxychloroquine in treating patients with advanced solid tumors that have not responded to chemotherapy	I	NCI-2012-03112
Cabozantinib	Cabozantinib or paclitaxel in treating patients with persistent or recurrent epithelial ovarian, fallopian tube, or primary peritoneal cavity cancer	II	NCI-2012-02058
AT13148	Phase I study of AT13148, a novel AGC kinase inhibitor	I	CRUKD/12/001
VS-6063	Phase I/Ib study of paclitaxel in combination with VS-6063 in patients with advanced ovarian cancer	I	VS-6063-101

Miscellanea			
MK-1775 (Wee-1 inhibitor)	Study with Wee-1 inhibitor MK-1775 and carboplatin to treat p53 mutated refractory and resistant ovarian cancer	II	M10MKO/MK1775-009
MK-1775 (Wee-1 inhibitor)	A study of MK-1775 in combination with paclitaxel and carboplatin versus paclitaxel and carboplatin alone for participants with platinum-sensitive ovarian tumors with the P53 gene mutation (MK-1775-004 AM4)	II	1775-004
BKM 120 (mutate PTEN; PIK3CA inhibitor)	BKM120 in advanced, metastatic, or recurrent endometrial cancers	II	HCI47841
BYL719 (PI3KNASE inhibitor)	A phase Ib/II study of the combination of BYL719 plus AMG 479 in adult patients with selected solid tumors	I/II	CBYL719X2105J
Cyclin dependent kinases 4 and 6	An open label study of the efficacy and safety of PD0332991, a selective inhibitor of the cyclin dependent kinases 4 and 6 in patients with recurrent ovarian cancer demonstrating Rb proficiency and Low p16 expression	II	11-003234
Triciribine (AKT inhibitor)	Triciribine and carboplatin in ovarian cancer	I/II	MCC-17035
LY2228820 (MAP KINASI)	A study of LY2228820 for recurrent ovarian cancer	I/II	12517
Inhibitor AKT (GSK2110183)	Dose-finding study on platinum-resistant ovarian cancer	I/II	PKB116611
MEK inhibitor	Trial of MEK inhibitor and PI3K/mTOR inhibitor in subjects with locally advanced or metastatic solid tumors	I	EMR 200066-006
MSC2015103B or pimasertib (MEK inhibitor)	MSC2015103B in solid tumors	I	EMR 200064-001
MEK162	A study of MEK162 and paclitaxel in patients with epithelial ovarian, fallopian tube, or peritoneal cancer	I	ARRAY-162-112
GSK2636771	A Phase I/IIa, first time in human, study of GSK2636771 in subjects with advanced solid tumors with phosphatase and tensin homolog (PTEN) deficiency	I/II	115717
OMP-54F28 (antagonist of the Wnt pathway	A dose escalation study of OMP-54F28 in subjects with solid tumors	I	54F28-001
